# ﻿*Impatiensbeipanjiangensis* (Balsaminaceae), a new species from Guizhou, China

**DOI:** 10.3897/phytokeys.241.113700

**Published:** 2024-04-30

**Authors:** Hong-Fen Hu, Jian Xu, Ming-Tai An, Ying Guo, Jia-Wen Yang

**Affiliations:** 1 College of Forestry, Guizhou University, CN-550025 Guiyang, China Guizhou University Guiyang China; 2 Guizhou Botanical Garden, CN-550000 Guiyang, China Guizhou Botanical Garden Guiyang China; 3 Natural Resources Bureau of Panzhou city, CN-553537 Panzhou, China Natural Resources Bureau of Panzhou city Panzhou China

**Keywords:** Balsaminaceae, Flora of China, morphology, phylogeny, taxonomy

## Abstract

*Impatiensbeipanjiangensis* Jian Xu & H. F. Hu (Balsaminaceae), a new species of Impatienssubg.Clavicarpa discovered in Guizhou, China, is described and illustrated in this study along with its molecular phylogenetic analysis. *I.beipanjiangensis* is similar to *I.liboensis*, *I.chishuiensis* and *I.clavigera* in morphology, but *I.tubulosa* has the closest relationship to it. However, there are various ways in which the new species can be easily distinguished from these four species: Inferior nodes swollen rhizoid, pale green and with hooked outer sepals, longer lateral united petals, subovate auricle, deeper lower sepal and shorter spur that is reflexed towards the lower sepal. Furthermore, *I.beipanjiangensis* is distinguished from other *Impatiens* species, based on morphological, micromorphological and palynological evidence and molecular data (PP 0.967).

## ﻿Introduction

The genus *Impatiens* L. is a member of the family Balsaminaceae, which contains over 1000 species worldwide, including approximately 270 species in China, primarily distributed in tropical and subtropical regions, such as Africa, India, south-western Asia, southern China and Japan, with a few species also being found in Europe, Siberia and northern China (Grey-Wilson 1980; Yu 2012; Li et al. 2022). *Impatiens* is characterised by stem fleshy, flowers bisexual and bisymmetric, with the lateral petals, spurred zygomorphic flowers with fused stamens surrounding the ovary and stigma and fruit an explosive capsule (Ruchisansakun et al. 2015). The genus, however, has become notoriously difficult for species classification and identification due to its complex morphological traits, small and endemic areas of occurrences, difficult preservation of plant specimens and difficult phylogenetic resolution at the infrageneric level (Li et al. 2022).

A new taxonomic system of Balsaminaceae with two subgenera (*Impatiens* and *Clavicarpa*), based on morphological and molecular evidence, was recently proposed by Yu et al. (2016) which has taxonomic implications for the Balsaminaceae definition (Ruchisansakun et al. 2021). The Impatienssubg.Clavicarpa has approximately 30 species, the majority of which are found in south-western China, particularly in Guangxi and Yunnan and share 8 key characteristics: racemes more than five flowers, four fully-developed lateral sepals, triangular 3-colpate pollen, 4-carpellate, clavate fruits, one ovule per carpel, elliptic seeds and seed coat with basic reticulate ornamentation (Kuang et al. 2014; Xia et al. 2019).

Southwest China is the distribution centre of the Impatienssubg.Clavicarpa, with Guangxi and Yunnan being areas where numerous explorations have been conducted for Balsaminaceae in recent years, resulting in a number of new species discoveries (Xia et al. 2019; Qin et al. 2020). In contrast, most areas of Guizhou still need to be investigated. In October 2019, during a field trip in Panzhou City, Guizhou Province, we found a new species of Balsaminaceae. We concluded that this species belongs to the Impatienssubg.Clavicarpa, but does not match any of the already described species, after extensive morphological comparison and phylogenetic analysis.

## ﻿Methods

### ﻿Gross morphology

The morphological characters of the new species, such as leaves, flowers and fruits, were carefully observed and measured in the field, with the plants subsequently being returned to the lab for detailed analysis. The specimens were then compared to the specimens available online, namely Kew Herbarium Catalogue (http://apps.kew.org/herbcat/navigator.do), Chinese National Herbarium (PE) (https://pe.ibcas.ac.cn/index.html) and JSTOR Global Plants (http://plants.jstor.org/). At the same time, specimens from online plant herbaria, such as China Virtual Herbarium (CVH) (https://www.cvh.ac.cn/index.php), Royal Botanical Garden Edinburgh (RBGE) (https://data.rbge.org.uk/search/herbarium/) and Chinese Academy of Sciences (KUN) (http://www.ui92.com/demo/html/1621/) were examined. Furthermore, the similar species, *I.liboensis*, *I.chishuiensis*, *I.clavigera* and *I.tubulosa*, were carefully observed and compared after the preliminary identification of the new species.

### ﻿Pollen grains and seeds

Mature, whole pollen grains and seeds collected from the field were observed directly and measured under magnification using an anatomical lens. Subsequently, they were mounted on double-sided adhesive tape and coated with a layer of gold before being photographed using a Hitachi SU8100 SEM. The micro-morphological characters were described following Wang and Wang (1983) and Lu (1991) for pollen grains and Lu and Chen (1991), Liu et al (2004) and Song et al (2005) for seeds. The average size of pollen grains and seeds was calculated, based on 20 of each.

### ﻿Taxon sampling and DNA sequencing

DNA sequences of the ITS marker from 150 species of Balsaminaceae were used for phylogenetic analysis, based on prior research (Yuan et al. 2004; Janssens et al. 2006; Yu et al. 2016), including two individuals of the putative new species, 148 species of *Impatiens* and one outgroup species (*Hydroceratriflora*). All sequences employed in this study were downloaded from GenBank, except *I.beipanjiangensis* which was newly generated for this study and species names and GenBank accession numbers are listed in Suppl. material 1.

The new species were sequenced using the ITS molecular marker (ITS-1 and ITS-4). The plant DNA isolation kit was used to extract DNA from fresh leaves using the Sangon Biotech Ezup column plant genomic DNA extraction kit (B518261). By amplified sequencing, agarose electrophoresis and gel recovery, the amplified PCR products were detected and purified. Sequencing reactions were carried out using an ABI Prism Bigdye Terminator Cycle Sequencing Kit (Applied Biosystems, Foster City, CA, USA). The products were analysed on an ABI3730xl automated DNA sequencer. All DNA samples were sent to Sangon Biotech (Shanghai) Co., Ltd. for sequencing.

### ﻿Phylogenetic analysis

Bayesian Inference (BI) was employed to infer the phylogenetic relationships in this study and all sequences were processed using Phylosuite v.1.2.3 (Zhang et al. 2019; Xiang et al. 2023). The sequence was aligned with MAFFT 7.313 (Katoh and Standley 2013) and the best model for BI was K2P + G4 (Kalyaanamoorthy et al. 2017) using the ModelFinder v.2.2.0 tool. Finally, BI analyses were conducted using MrBayes 3.2.7a on the ITS dataset (Ronquist et al. 2012). The resulting trees with node support values was visualised on the chiplot website (https://www.chiplot.online/).

## ﻿Results

### ﻿ Impatiens beipanjiangensis

Taxon classificationPlantaeEricalesBalsaminaceae

Jian Xu & H.F. Hu sp. nov.

B148A56D-BB05-5CC1-98BC-AEA7355FAD61

urn:lsid:ipni.org:names:77340983-1

Figs 1 , 2

#### Type.

China, Guizhou, Panzhou City, Pugu Township, humid valley, alt. 1306 m, 26°3'13.66"N, 104°44'33.93"E, 06 Oct 2019, *Jian Xu, Ying Guo and Jia-Wen Yang* (holotype: GZAC!20191006PZ001, isotype: GZAC!20191006PZ002).

#### Diagnosis.

This species is similar in morphology to *I.liboensis*, *I.chishuiensis* and *I.clavigera* and is close in phylogeny to *I.tubulosa*. Their leaf margin with crenate and fimbriae, raceme, stamens 5, ovary clavate, capsule hammer-shaped. However, it is different in the following aspects: inferior nodes swollen rhizoid, pale green and with hooked outer sepals, longer lateral united petals, subovate auricle, deeper lower sepal and shorter spur that is reflexed towards the lower sepal.

**Figure 1.**
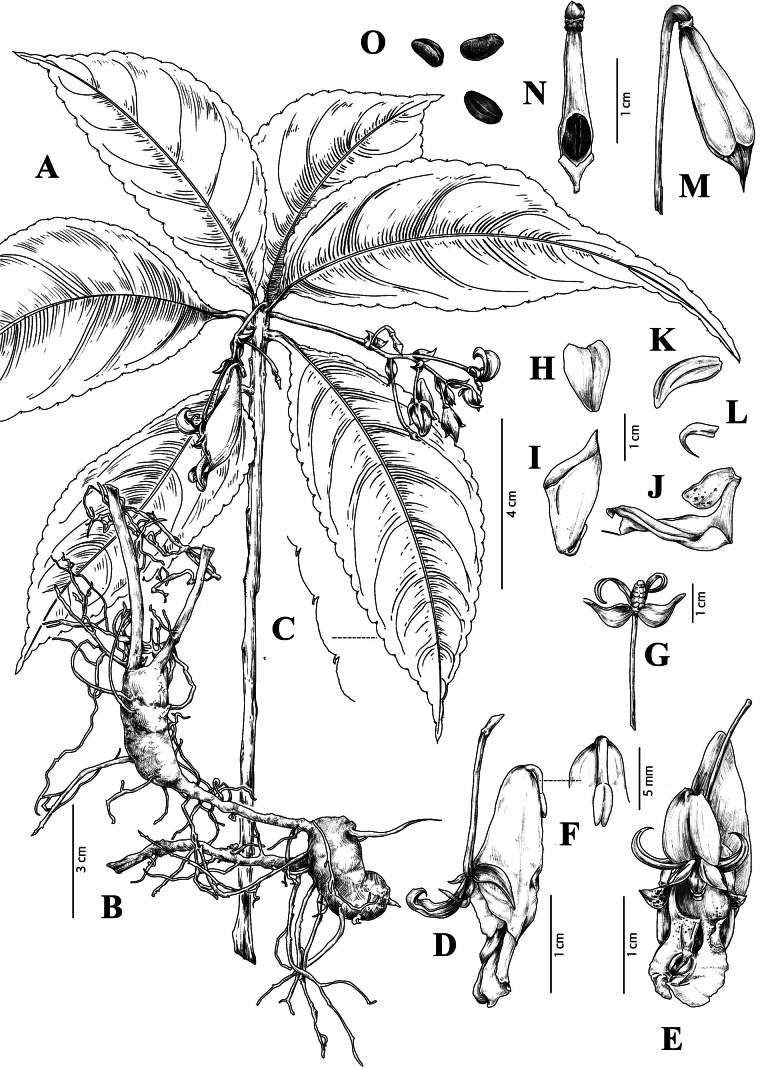
*Impatiensbeipanjiangensis* Jian Xu & H.F. Hu, sp. nov. **A** whole plant **B** root **C** margin of leaf**D** flower in side view**E** flower in front view **F** spur **G** lateral sepals **H** dorsal petal **I** inner lateral sepal **J** lateral united petals **K** inner lateral sepal **L** outer lateral sepal **M** fruit **N** fruit anatomy **O** seeds (Drawn by Gan-Yang Yu from Guizhou University).

#### Description.

Plants perennial, 40–70 (110) cm tall, glabrous. Procumbent rhizome, inferior nodes swollen, nodes 3–5 cm long and 1–3 cm wide; stem erect, unbranched, fleshy. Leaves alternate, often dense in the upper part of the stem, membranous, elliptic or elliptic-lanceolate, 7–15 cm long, 2.5–4.5 cm wide, deep green above, pale green beneath, apex acuminate, base cuneate; margin obtusely crenate, with fimbriae between teeth, lateral veins 5–7 pairs; petioles 0.5–2 cm long, without glands. Racemes in upper leaf axils, 5–7 flowers, a bract at base, ovate, 0.7–1 cm long, persistent. Peduncle 5–7 cm long. Pedicels 1.5–3 cm long. Flowers yellow, with red spots or not, 3–5 cm long. Lateral sepals 4, pale green, semi-transparent; the outer 2 oblique ovate, outwards hooked to dorsal petal, 1.1–1.3 cm long, 0.5–0.7 cm wide, unequal sides, longitudinally 4–5 veined, apex inwardly curved; the inner 2 linear-lanceolate, 1–1.3 cm long, 0.1–0.3 cm wide, apex recurved. Dorsal petal obovate, 1–1.5 cm long, 0.9–1 cm wide, abaxial mid-vein narrowly carinate. Lateral united petals not clawed, connate, 2–lobed, 2.3–2.8 cm long; basal lobes oblique ovate, 0.9–1.1 cm long, apex acute; lower lobes, elliptic, 2–2.3 cm long, slightly retuse at apex, with a abaxial auricle inflexed, subovate. Lower sepal infundibuliform, 4–5 cm deep, mouth oblique, 1.8–2.3 cm wide, apex acute, gradually constricted to a reflexed and short spur at base; spur 0.8–1.2 cm long, apex bilobed, reflexed towards lower sepal. Stamens 5, anthers small and white, apex obtuse; filaments linear, 2.8–3.3 cm long. Ovary clavate, 4-carpellate, fusiform, ca. 0.5 cm long. Capsule hammer-shaped, 1.4–1.8 cm, superior part inflated, apex mucronulate. Seeds 4, narrowly ellipsoid, dark brown, 0.38–0.43 cm long, 0.19–0.22 cm wide.

**Figure 2.**
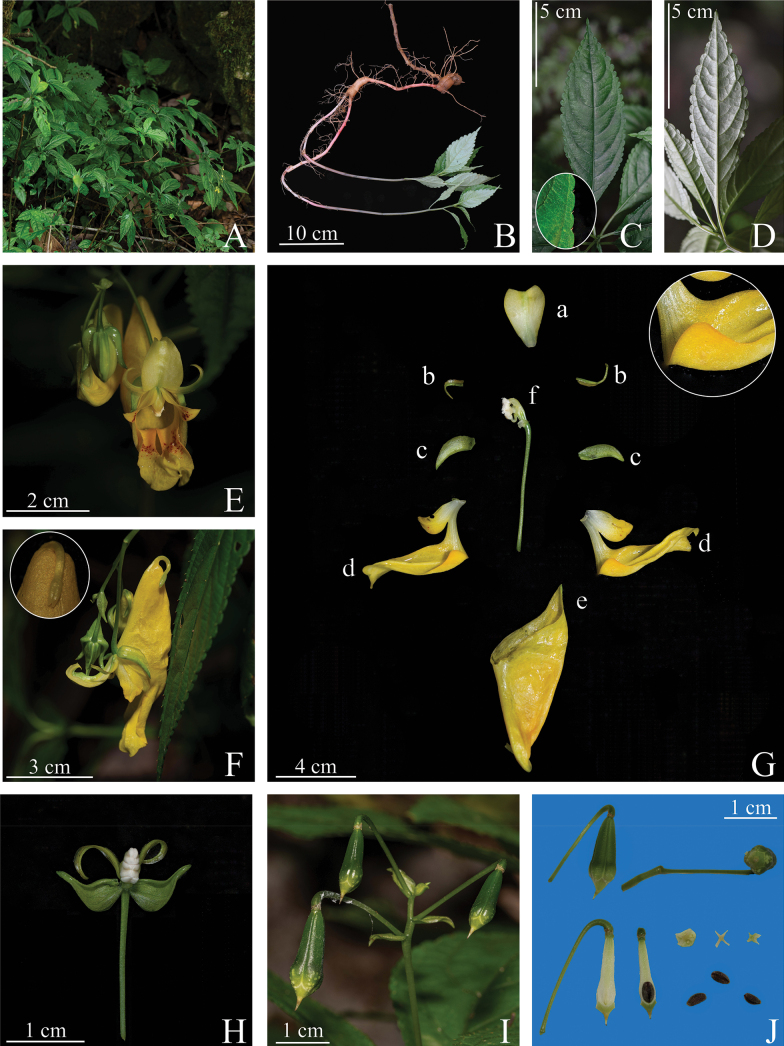
*Impatiensbeipanjiangensis* Jian Xu & H.F. Hu, sp. nov. **A** habitat **B** whole plant and root **C** adaxial surface of leaf blade and margin of leaf (inset) **D** abaxial surface of leaf blade **E** flower in front view **F** flower in side view and spur (inset) **G** flower anatomy and auricle (inset) (a) dorsal petal (b) outer lateral sepals (c) inner lateral sepals (d) lateral united petals (e) lower sepal (f) filaments and anthers **H** lateral sepals **I** fruit **J** fruit anatomy and seeds (Photographed by Jian Xu).

#### Phenology.

Flowering October to November, fruiting November to December.

#### Etymology.

The specific epithet “beipanjiangensis” refers to the river basin of the type specimen, Panzhou City (Beipanjiang River Basin), Guizhou, China.

#### Vernacular name.

The Chinese name is “Bēi Pán Jiāng Fèng Xiān Huā” (北盘江凤仙花).

#### Distribution and habitat.

**Figure 3.**
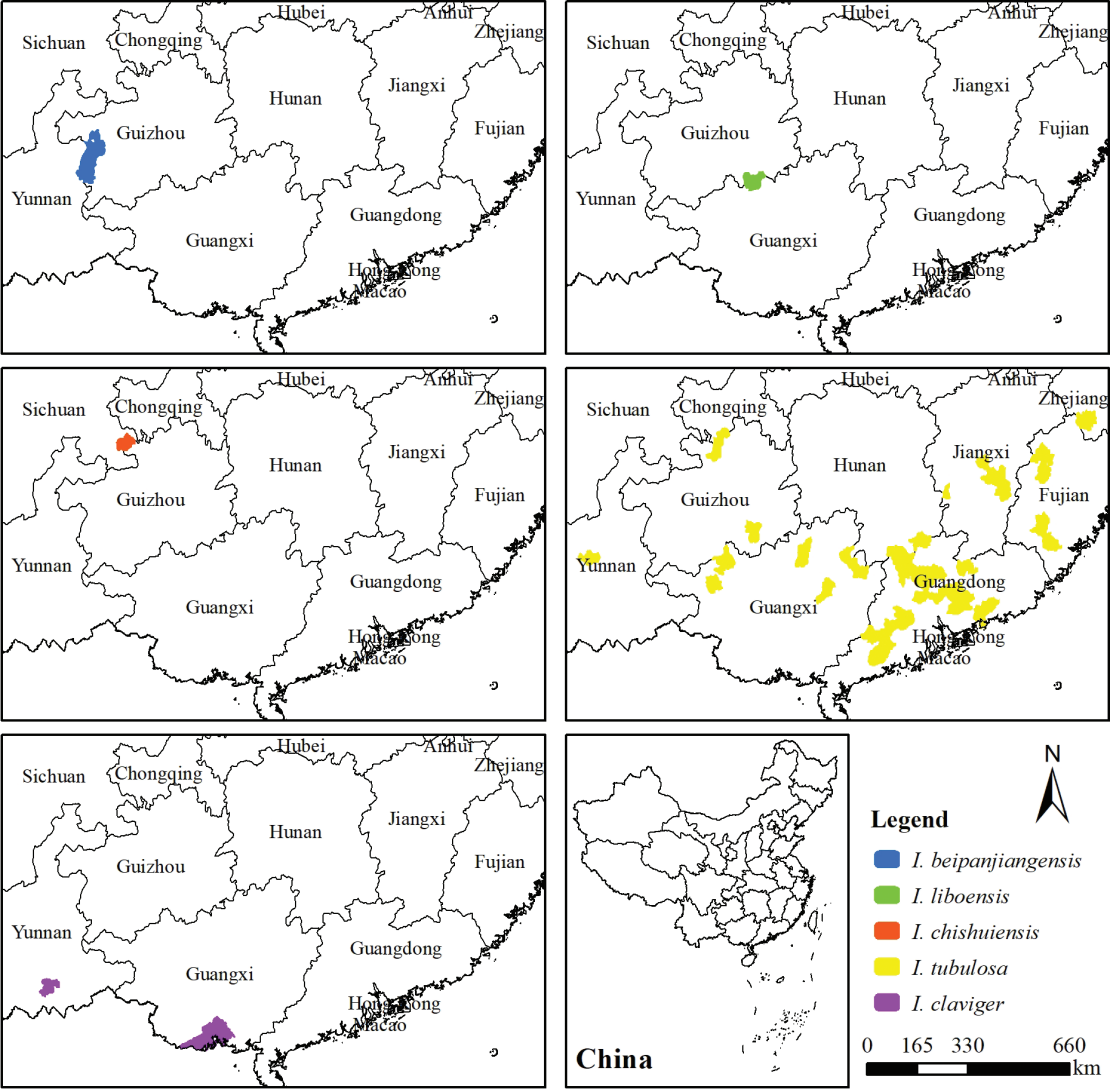
Geographical distribution of *I.beipanjiangensis, I.liboensis, I.chishuiensis, I.tubulosa* and *I.clavigera*.

This species is only known to be found in western Guizhou (Fig. 3), growing in the humid valley at alt. 1300–1500 m. Type specimens were collected from Panzhou City (Beipanjiang River Basin), Guizhou, China. At present, four populations of *I.beipanjiangensis* have been found in Pugu Township, Panzhou City. In addition, a population of *I.beipanjiangensis* was found in Huajia Township, Shuicheng District.

#### Conservation status.

According to the current distribution of the population, we provisionally assess its status as Endangered [EN], based on criterion B2ab[i,ii], with the five distribution points and AOO of 20 km^2^, within the range of “Endangered” status(IUCN 2012). Furthermore, there are about 5100 individuals in the five distribution points and EOO is 12190 km^2^.

#### Additional specimens examined.

China, Guizhou, Panzhou City, Pugu Township, humid valley, alt. 1487 m, 26°3'42.55"N, 104°44'30.47"E, 06 Oct 2019, Jian Xu, Ying Guo and Jia-Wen Yang (paratype: GZBG!XJ20191006001, isotype: GZBG!XJ20191006001).

#### Similar species.

**Figure 4.**
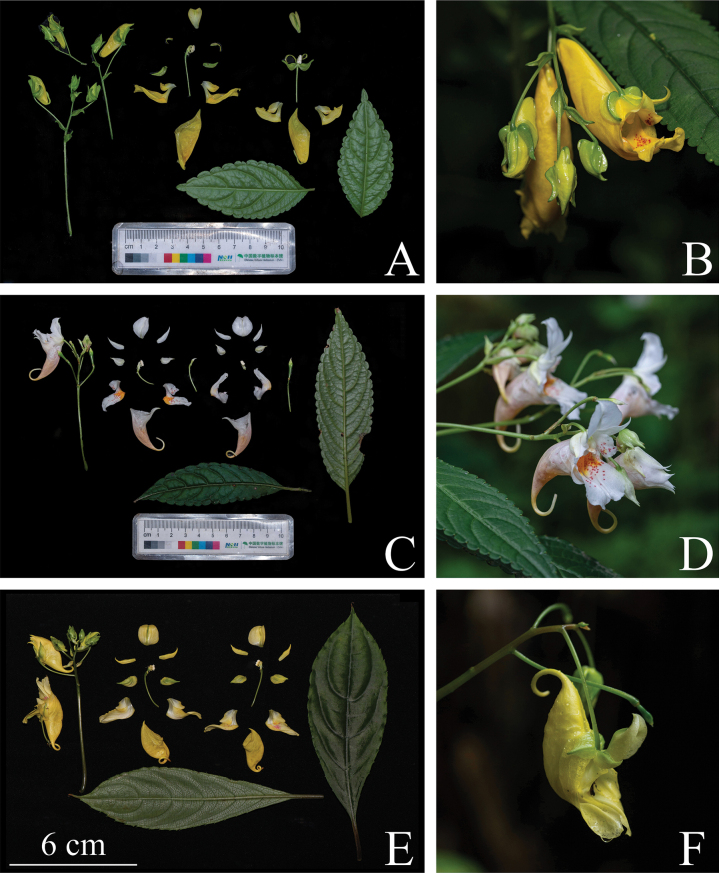
Detailed comparison diagram of *Impatiensbeipanjiangensis*, *I.liboensis* and *I.chishuiensis***A** flower anatomy of *I.beipanjiangensis***B** flower of *I.beipanjiangensis***C** flower anatomy of *I.liboensis***D** flower of *I.liboensis***E** flower anatomy of *I.chishuiensis***F** flower of *I.chishuiensis* (Photographed by Jian Xu).

*I.beipanjiangensis* is similar to *I.liboensis, I.chishuiensis* and *I.clavigera* in morphology, but *I.tubulosa* has the closest relationship to it. However, after careful comparison, we found that the five species were significantly different in life form, root, outer lateral sepals, lateral united petals, lower sepal, pollen, seed and other traits. Additionally, the traits expression of each population of the new species was relatively stable and there was no significant difference. Deleted morphological comparison of four species is shown in Table 1 and Fig. 4:

**Table 1.** Detailed comparison of *I.beipanjiangensis*, *I.liboensis*, *I.chishuiensis*, *I.tubulosa* and *I.clavigera*.

Characters	*I.beipanjiangensis*	*I.liboensis*	*I.chishuiensis*	*I.tubulosa*	*I.clavigera*
Life form	perennial	perennial	annual	annual	perennial
Plant height	40–70(10) cm	22–50 cm	30–50 cm	30–40 cm	50–60 cm
Root type	rhizome, inferior nodes swollen	underground tuber	rhizome, not swollen	fibrous root	rhizome, not swollen
Petiole	0.5–2 cm	1.5–5 cm	0.5–2 cm	0.5–1.5 cm	1–2 cm
Flowers colour	yellow	white or pink-white	yellow	yellow or yellow-white	pale yellow
Outer lateral sepals	pale green, oblique ovate, hooked curved, 1.1–1.3 cm long	white, oblique ovate, 0.9–1 cm long	yellow, oblique ovate or ovate, 0.9–1 cm long	white, oblique ovate, 0.5–0.6 cm long	yellow green, oblique ovate, 0.8–1.2 cm long
Lateral united petals	not clawed, 2.3–2.8 cm long; lower lobes elliptic, slightly retuse at apex, with a abaxial auricle inflexed, subovate	not clawed, ca. 2 cm long; lower lobes obovate-oblong or obliquely obovate, slightly retuse at apex, with a abaxial auricle inflexed, suborbicular	not clawed, ca. 2.5 cm long; lower lobes oblong, apex obtuse, with a abaxial auricle inflexed, suborbicular	shortly clawed, ca. 1.5 cm long, lower lobes obovate, apex obtuse, auricle absent	not clawed, 2.5–2.6 cm long; lower lobes oblong, with a abaxial auricle, round,
Lower sepal	infundibuliform, 4–5 cm deep; mouth oblique, 1.8–2.3 cm wide	saccate, 2.5–3 cm deep; mouth vertical, 2.5–3 cm wide	broadly infundibuliform, ca. 2 cm deep; mouth vertical, ca. 2 cm wide	saccate, 2–2.5 cm deep; mouth lightly oblique, ca. 1.5 cm wide	deeply saccate, ca. 3 cm deep; mouth oblique, ca. 2 cm wide
Spur	0.8–1.2 cm long, apex bilobed, reflexed towards the lower sepal	0.8–1.2 cm long, apex bilobed, incurved	1.5–1.8 cm long, apex acuminate, incurved	ca. 2 cm long, apex acuminate, recurved	ca. 1 cm long, apex acuminate, incurved

#### Palynology and seed description.

**Figure 5.**
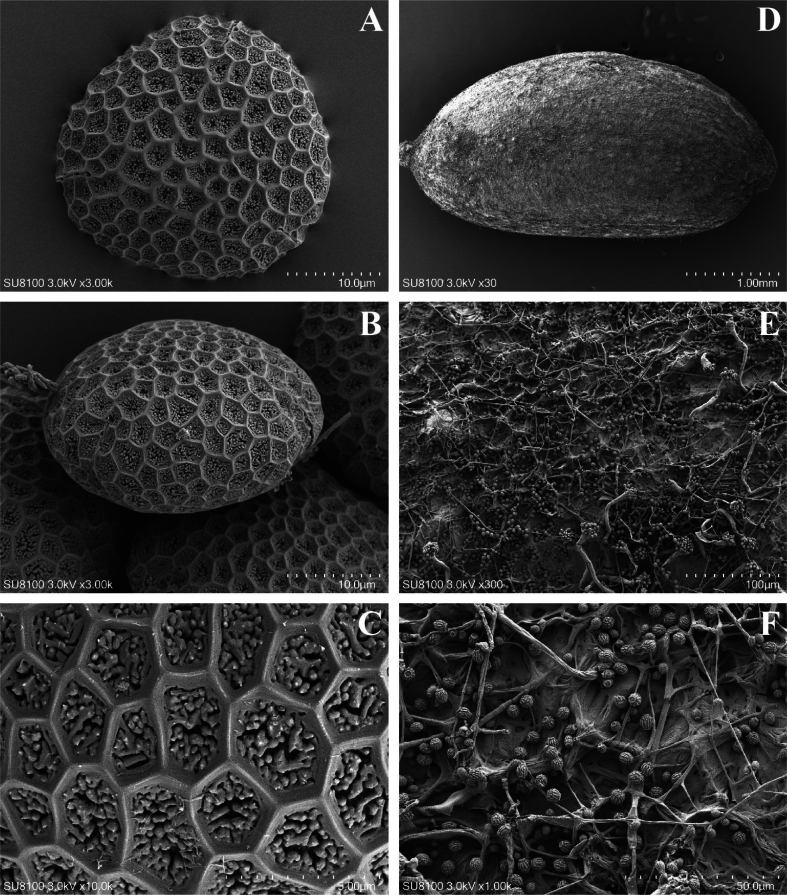
Scanning electron microscope images of seeds and pollen grains of *Impatiensbeipanjiangensis* Jian Xu & H.F. Hu, sp. nov. **A–C** pollen grains of *I.beipanjiangensis***D–F** seeds of *I.beipanjiangensis*.

The pollen grains of *I.beipanjiangensis* are triangular in polar view, tricolpate with long, thin colpi, an exine with reticulate ornamentation and dense granules in lumina (Fig. 5A–C). The average size of Polar length × equatorial length (P × E) is 19.44 (18.42–20.36) × 31.12 (29.61–32.42) μm. The seeds of *I.beipanjiangensis* are 0.38–0.43 cm × 0.19–0.22 cm, dark brown in colour, narrowly ellipsoid. The seed surface with reticulate ornamentation and the shape is very irregular. The meshes are slightly sunken, the bottoms are wrinkled and there are ellipsoidal appendages in the mesh. The reticular ridges are folded and top curved (Fig. 5D–F). The seed morphology of *I.beipanjiangensis* is different from other species of Impatiens in size and appendages. The pollen morphology of *I.beipanjiangensis* is also quite different from its related species (Zeng et al. 2016). The detailed comparison is shown in Table 2.

**Table 2.** Detailed palynology comparison of *Impatiensbeipanjiangensis*, *I.liboensis*, *I.chishuiensis*, *I.tubulosa* and *I.clavigera*.

Species	Shape in polar view	Polar length × equatorial length (P × E)/um	Granules in lumina
*I.beipanjiangensis*	triangular	19.44 (18.42–20.36) × 31.12 (29.61–32.42)	++++
*I.liboensis*	triangular-subcircular	15.3 (14.8–15.5) × 39.2 (38.8–40.6)	+++
*I.chishuiensis*	subellipsoid	27.0–29.2 × 25.8–26.5	+++++
*I.tubulosa*	triangular	13.3 (14.8–15.5) × 36.2 (34.7–37.6)	+++
*I.clavigera*	triangular-subcircular	32.4 (30.5–32.9) × 36.8 (32.5–37.3)	++++

Note: “+” represents granule density in the lumina.

#### Molecular phylogenetic evidence.

**Figure 6.**
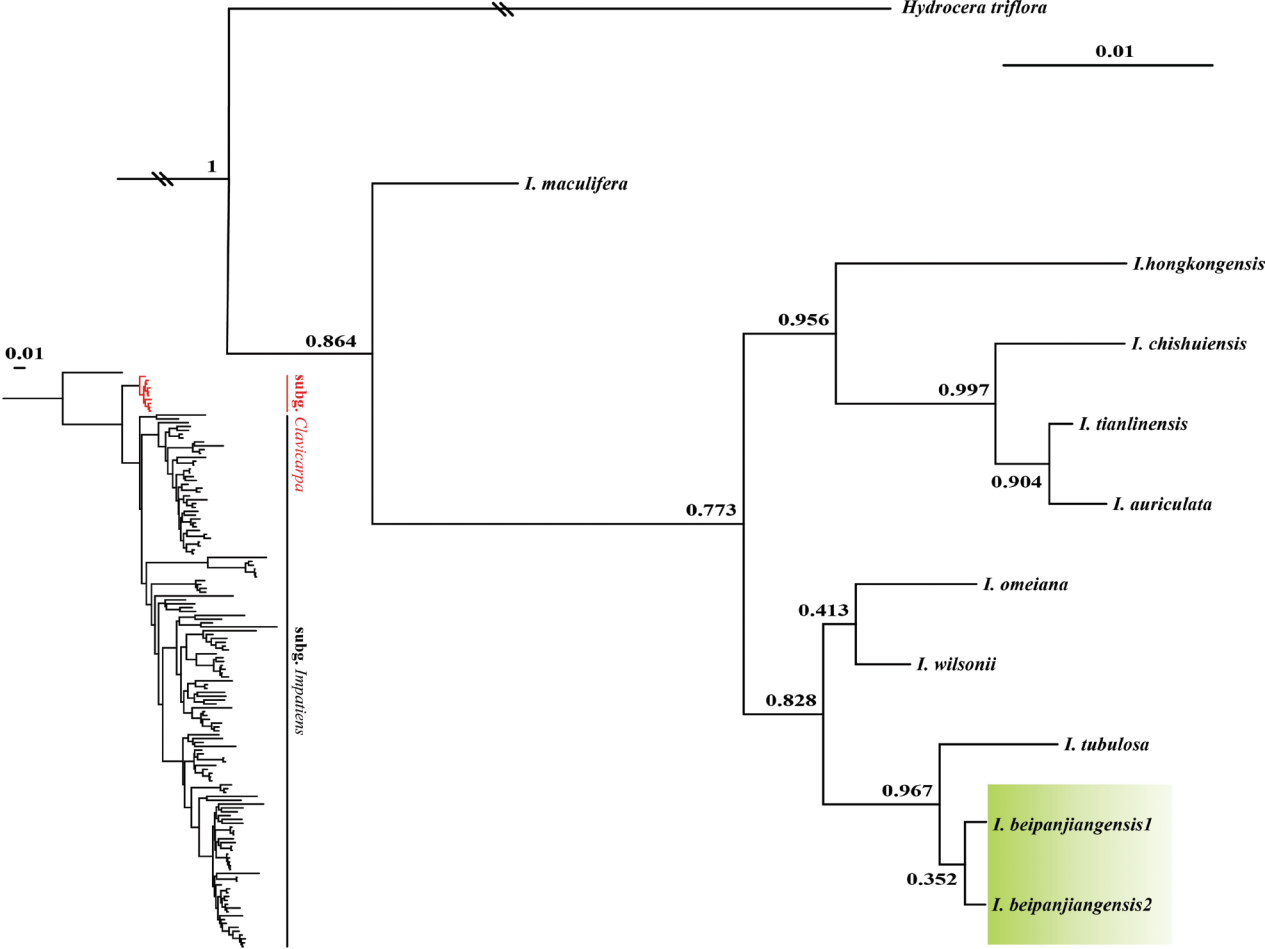
Partial Bayesian consensus phylogram, based on ITS sequences. Numbers above and below branches are Bayesian posterior probabilities.

In the phylogenetic tree, based on the ITS sequences (Fig. 6), two sequences of *I.beipanjiangensis* were clustered together, indicating that it is a distinct new species in the Impatienssubg.Clavicarpa and *I.beipanjiangensis, I.tubulosa* and *I.wilsonii*, as well as *I.omeiana*, form a small clade. The sister species of *I.beipanjiangensis* is *I.tubulosa*; however, the strong support demonstrates the uniqueness of *I.beipanjiangensis* (PP 0.967). At the same time, the morphological features and phenological period of *I.beipanjiangensis* also reveal that this species is markedly different from *I.tubulosa* (Table 1). The Bayesian phylogenetic tree topology is similar to those obtained in previous studies (Tan et al. 2015; Xia et al. 2019; Qin et al. 2020; Ruchisansakun et al. 2021). The phylogenetic tree results further proved the distinctiveness of *I.beipanjiangensis*, which is congruent with the morphological comparison result.

## Supplementary Material

XML Treatment for Impatiens beipanjiangensis

## ﻿Additional information

### Author ORCIDs

Hong-Fen Hu https://orcid.org/0009-0008-4761-8242

Jian Xu https://orcid.org/0000-0002-0714-0917

Ming-Tai An https://orcid.org/0000-0003-3886-0287

### Data availability

All of the data that support the findings of this study are available in the main text or Supplementary Information.

## Supplementary materials

Supplementary material 1 The GenBank accession numbers for DNA sequences used in this study Data type doc https://binary.pensoft.net/file/1040277This dataset is made available under the Open Database License (http://opendatacommons.org/licenses/odbl/1.0/). The Open Database License (ODbL) is a license agreement intended to allow users to freely share, modify, and use this Dataset while maintaining this same freedom for others, provided that the original source and author(s) are credited.Hong-Fen Hu, Jian Xu, Ming-Tai An, Ying Guo, Jia-Wen Yang
