# Supplementary material for: ﻿Impatiensbeipanjiangensis (Balsaminaceae), a new species from Guizhou, China
Source: PhytoKeys. 2024 Apr 30;241:201–13. doi: 10.3897/phytokeys.241.113700 (PMC11077263; doi:10.3897/phytokeys.241.113700)
Supplement: Supplementary material 1 — The GenBank accession numbers for DNA sequences used in this study [file phytokeys-241-201_article-113700__-s001.doc]

**Appendix 1**

**Table A1.** The GenBank accession numbers for DNA sequences used in this study.

| **Species** | **GenBank Accession number( ITS)** |
| --- | --- |
| *Hydrocera triflora* | AY348853 |
| *Impatiens acehensis* | AY348739 |
| *Impatiens amoena* | AY348795 |
| *Impatiens amphorata* | AY348740 |
| *Impatiens andohahelae* | AY348741 |
| *Impatiens andringitrensis* | AY348742 |
| *Impatiens angulata* | KP776060 |
| *Impatiens anovensis* | AY348743 |
| *Impatiens aquatilis* | AY348745 |
| *Impatiens arguta* | AY348746 |
| *Impatiens aureliana* | AY348747 |
| *Impatiens auricoma* | AY348748 |
| *Impatiens auriculata* | MN095283 |
| *Impatiens balsamina* | AY348749 |
| *Impatiens barbata* | AY348750 |
| *Impatiens baronii* | AY348751 |
| *Impatiens begoniifolia* | AY348752 |
| *Impatiens bequaertii* | AY348753 |
| *Impatiens bicornuta* | AY348754 |
| *Impatiens blinii* | KP776063 |
| *Impatiens bombycina* | AY348755 |
| *Impatiens brachycentra* | AY348756 |
| *Impatiens burtonii* | AY348757 |
| *Impatiens campanulata* | AY348758 |
| *Impatiens capensis* | AY348759 |
| *Impatiens chekiangensis* | KP776064 |
| *Impatiens chinensis* | AY348761 |
| *Impatiens chishuiensis* | KP776065 |
| *Impatiens chiulungensis* | KP776066 |
| *Impatiens chlorosepala* | KP776067 |
| *Impatiens columbaria* | AY348764 |
| *Impatiens conchibracteata* | AY348765 |
| *Impatiens congolensis* | AY348766 |
| *Impatiens corchorifolia* | AY348767 |
| *Impatiens cordata* | AY348768 |
| *Impatiens cuspidata* | AY348769 |
| *Impatiens cyanantha* | AY348770 |
| *Impatiens cyathiflora* | AY348771 |
| *Impatiens cymbifera* | KP776069 |
| *Impatiens davidii* | KP776070 |
| *Impatiens delavayi* | AY348773 |
| *Impatiens desmantha* | AY348774 |
| *Impatiens drepanophora* | AY348776 |
| *Impatiens duclouxii* | KP776071 |
| *Impatiens eubotrya* | AY348777 |
| *Impatiens faberi* | AY348778 |
| *Impatiens falcifer* | KP776072 |
| *Impatiens firmula* | AY348780 |
| *Impatiens fischeri* | AY348781 |
| *Impatiens fissicornis* | AY348782 |
| *Impatiens flanaganae* | AY348783 |
| *Impatiens forrestii* | AY348784 |
| *Impatiens fragicolor* | KP776073 |
| *Impatiens fuchsioides* | AY348785 |
| *Impatiens furcata* | AY348786 |
| *Impatiens gongshanensis* | KP776074 |
| *Impatiens harae* | KP776075 |
| *Impatiens henslowiana* | AY348790 |
| *Impatiens hians* | AY348791 |
| *Impatiens hoehnelii* | AY348792 |
| *Impatiens holocentra* | AY348793 |
| *Impatiens hongkongensis* | KP776076 |
| *Impatiens hunanensis* | KP776077 |
| *Impatiens imbecilla* | AY348796 |
| *Impatiens inaperta* | AY348797 |
| *Impatiens keilii* | AY348798 |
| *Impatiens kerriae* | AY348799 |
| *Impatiens kilimanjari* | AY348800 |
| *Impatiens lateristachys* | KP776078 |
| *Impatiens laxiflora* | KP776079 |
| *Impatiens lecomtei* | AY348802 |
| *Impatiens leptocaulon* | KP776080 |
| *Impatiens leptopoda* | AY348787 |
| *Impatiens leschenaultii* | AY348803 |
| *Impatiens levingei* | AY348804 |
| *Impatiens lobulifera* | KP776081 |
| *Impatiens mackeyana subsp. claeri* | AY348763 |
| *Impatiens mackeyana subsp. zenkeri* | AY348852 |
| *Impatiens macrovexilla* | KP776082 |
| *Impatiens maculifera* | MN095280 |
| *Impatiens manaharensis* | AY348805 |
| *Impatiens margaritifera* | KP776084 |
| *Impatiens mengtszeana* | AY348806 |
| *Impatiens meruensis* | AY348807 |
| *Impatiens microstachys* | KP776085 |
| *Impatiens miniata* | AY348809 |
| *Impatiens monticola* | AY348810 |
| *Impatiens morsei* | KP776086 |
| *Impatiens napoensis* | AY348811 |
| *Impatiens neglecta* | KP776087 |
| *Impatiens niamniamensis* | AY348812 |
| *Impatiens noli-tangere* | KP776088 |
| *Impatiens nubigena* | KP776089 |
| *Impatiens nyimana* | KP776090 |
| *Impatiens obesa* | KP776091 |
| *Impatiens omeiana* | KC905505 |
| *Impatiens oxyanthera* | AY348814 |
| *Impatiens pandurata* | KU042074 |
| *Impatiens parasitica* | AY348815 |
| *Impatiens parviflora* | AY348816 |
| *Impatiens percrenata* | AY348817 |
| *Impatiens pingxiangensis* | KP776093 |
| *Impatiens piufanensis* | KP776094 |
| *Impatiens platychlaena* | AY348818 |
| *Impatiens platypetala* | AY348819 |
| *Impatiens platysepala* | KP776095 |
| *Impatiens poculifer* | AY348820 |
| *Impatiens principis* | KP776096 |
| *Impatiens pritzelii* | AY348821 |
| *Impatiens pseudoviola* | AY348822 |
| *Impatiens pterosepala* | KP776097 |
| *Impatiens purpurea* | AY348823 |
| *Impatiens racemosa* | KP776098 |
| *Impatiens radiata* | AY348824 |
| *Impatiens rectangula* | AY348825 |
| *Impatiens rothii* | AY348827 |
| *Impatiens rubrostriata* | AY348828 |
| *Impatiens sambiranensis* | AY348829 |
| *Impatiens scabrida* | KP776099 |
| *Impatiens scullyi* | KP776100 |
| *Impatiens scutisepala* | AY348830 |
| *Impatiens siculifer* | KP776101 |
| *Impatiens sodenii* | AY348832 |
| *Impatiens soulieana* | AY348833 |
| *Impatiens stenosepala* | AY348835 |
| *Impatiens stuhlmannii* | AY348836 |
| *Impatiens subabortiva* | AY348837 |
| *Impatiens sulcata* | KP776103 |
| *Impatiens sunkoshiensis* | KP776104 |
| *Impatiens taronensis* | AY348838 |
| *Impatiens tayemonii* | AY348839 |
| *Impatiens teitensis* | AY348840 |
| *Impatiens textorii* | AY348841 |
| *Impatiens tianlinensis* | KT321312 |
| *Impatiens tienmushanica* | KP776105 |
| *Impatiens tortisepala* | KP776106 |
| *Impatiens trichosepala* | AY348843 |
| *Impatiens tuberculata* | KP776107 |
| *Impatiens tuberosa* | AY348844 |
| *Impatiens tubulosa* | KP776108 |
| *Impatiens uliginosa* | AY348845 |
| *Impatiens urticifolia* | KP776109 |
| *Impatiens usambarensis* | AY348847 |
| *Impatiens vilersi* | AY348848 |
| *Impatiens walleriana* | AY348849 |
| *Impatiens wenshanensis* | KP776110 |
| *Impatiens wilsonii* | KP776111 |
| *Impatiens xanthina* | AY348850 |
| *Impatiens yaoshanensis* | KP776112 |
